# Immunophenotype and Immune-Modulatory Activities of Human Fetal Cartilage-Derived Progenitor Cells

**DOI:** 10.1177/0963689719842166

**Published:** 2019-04-14

**Authors:** Su Jeong Lee, Jiyoung Kim, Woo Hee Choi, So Ra Park, Byung Hyune Choi, Byoung-Hyun Min

**Affiliations:** 1 Department of Molecular Science and Technology, Ajou University, Suwon, Korea; 2 Department of Physiology, Inha University College of Medicine, Incheon, Korea; 3 Department of Biomedical Sciences, Inha University College of Medicine, Incheon, Korea; 4 Cell Therapy Center, Ajou University Medical Center, Suwon, Republic of Korea; 5 Department of Orthopaedic Surgery, School of Medicine, Ajou University, Suwon, Korea

**Keywords:** human fetal cartilage-derived progenitor cells, immunogenicity, immunomodulation, cell therapy

## Abstract

We have previously reported human fetal cartilage progenitor cells (hFCPCs) as a novel source of therapeutic cells showing high proliferation and stem cell properties superior to those of adult mesenchymal stem cells (MSCs). In this study, we investigated the immunophenotype and immune-modulatory activities of hFCPCs. With institutional review board approval, hFCPCs were isolated from fetuses at 11–13 weeks of gestation. hFCPCs showed strong expression of HLA class I molecules but low or no expression of HLA class II and co-stimulatory molecules, which was not changed significantly after 4 days of IFN-γ treatment. In a mixed lymphocyte reaction (MLR), hFCPCs showed no allogeneic immune response to peripheral blood lymphocytes (PBLs) and suppressed concanavalin A (Con A)-mediated proliferation of PBLs in a dose-dependent manner. In addition, hFCPCs inhibited Con A-induced secretion of pro-inflammatory cytokines TNF-α and IFN-γ from PBLs but showed no significant decrease of secretion of IL-10, anti-inflammatory cytokine. Co-culture of hFCPCs with stimulated PBLs for 4 days resulted in a significant increase in CD4^+^CD25^+^FoxP3^+^ T regulatory cells (Tregs). hFCPCs expressed LIF, TGF-β1, TSG-6, and sHLA-G5 but did not express IDO and HGF. Stimulation of hFCPCs with TNF-α for 12 h showed slight induction in the expression of LIF, TSG-6, IDO, and HGF, whereas stimulation with IFN-γ did not affect expression of any of these factors. These results suggest that hFCPCs have low allogeneic immunogenicity and immune-modulatory activity *in vitro*, comparable to those of MSCs. However, compared with MSCs, hFCPCs were less responsive to TNF-α and IFN-γ, and the mechanisms underlying responses to these two cell types appeared distinct.

## Introduction

Mesenchymal stem cells (MSCs) are an attractive source of cells for therapeutic use, because they are easy to obtain from several tissues in adults, have low immunogenicity and chemotactic activity, and can differentiate into a series of mesengenic cell lineages. The ability of MSCs to modulate immune responses and inflammation *in vitro* and *in vivo* raised considerable interest because of their potential for use in treating many immune-related diseases^1^. However, MSCs have an insufficient differentiation ability, limiting their potential to meet clinical needs for tissue regeneration, and they show phenotypic drift during long-term expansion, hindering their mass production. Studies are currently underway to overcome these practical limitations of MSCs, but there is also a keen demand to find a novel source of cells. Embryonic stem cells (ESCs) and induced pluripotent stem cells (iPSCs) are good sources of therapeutic cells, but there are high safety concerns and technical challenges associated with their use, and these cells do not have immune-privilege and immune-modulatory functions^2,3^.

In contrast, stem or progenitor cells from fetal tissues may complement, or be a substitute for, MSCs. They can be isolated from a variety of different fetal tissues, including bone marrow, liver, blood^4^, lung^5^, brain^6^, cartilage^7^, heart^8^, umbilical cord blood^9^, Wharton’s jelly^10^, and placenta^11^. Fetal stem/progenitor cells have a greater proliferative capacity and differentiation potential than MSCs^12^. In addition, they have the advantages of low tumorigenicity and immunogenicity^13,14,15^. Several studies have shown that fetal stem/progenitor cells have an immune-modulatory activity similar to those of MSCs^14,15^. However, most of the studies have been done using post-natal placenta or umbilical cord blood-derived MSCs and immune-modulatory activity of MSCs from pre-natal fetus is limited. In addition, it is not clear what the differences are between the immune-modulatory activity of selected subpopulation of MSCs and total fetal progenitor cells. Therefore, it is imperative to understand the immune characteristics and immune-modulatory functions of cells from many different fetal tissues for their clinical adoption.

Many previous studies have established the mechanism of immune-privileged and immune-modulatory abilities of MSCs. MSCs express MHC class I molecules but do not express HLA class II molecules and co-stimulatory factors such as CD80, CD86, and CD40^16^. Functional assays show that MSCs inhibit proliferation of T and B lymphocytes^17^, reduce cytotoxicity of T lymphocytes^18,19^ and natural killer cells^18^, suppress differentiation and maturation of monocytes into dendritic cells^20^, and stimulate production of T regulatory cells (Tregs) from immature T cells^21^. Many cytokines and ligands secreted by MSCs are known to modulate these processes, including interleukin 10 (IL-10)^22^, leukemia inhibitory factor (LIF)^19^, indoleamine 2,3-dioxygenase (IDO)^18,23^, prostaglandin E2 (PGE2)^18^, hepatocyte growth factor (HGF)^24^, transforming growth factor (TGF)-β1^24^, soluble human leukocyte antigen-G5 (sHLA-G5)^25^, and TNF-α stimulated gene 6 (TSG-6)^26^.

Fetal tissues are immune tolerant to limit their reactions to the mother^27^. They show low level expression of HLA class I and co-stimulatory molecules, and produce immune modulatory molecules such as TGF-β^13^. The mechanisms of immune tolerance involve stimulation of CD4^+^CD25^+^FoxP3^+^ Tregs and auto-reactive T cell clones from the thymus^28^. MSCs are also found in some fetal tissues, such as those of the liver^14^ and bone marrow^29^, and they show low immunogenicity, immune-modulatory activity, and inflammatory cytokine secretion, similar to adult MSCs. Fetal neural progenitor cells (NPCs)^30^ and fetal bone cells^13^ are also reported to have similar immuno-phenotypes, but not much information is available on other fetal tissues. Interestingly, these two fetal tissue-derived cells exhibit differential expression patterns of HLA class I and II molecules in an unstimulated state and upon stimulation with TNF-α and IFN-γ. Fetal NPCs express higher levels of HLA class I molecules than class II molecules, and neither of these was induced by treatment with TNF-α or INF-γ. In contrast, fetal bone cells show very low expression of both HLA class I and II molecules, but their expression increases significantly in response to TNF-α or INF-γ. These results suggest that immune-related activities of fetal stem/progenitor cells might vary with tissue type, and these differences need to be clarified for therapeutic development of each cell type.

Previously, we isolated human fetal cartilage progenitor cells (hFCPCs) from a fetus at 12 weeks of gestation and identified their stem cell properties of high colony formation and proliferation, and multi-potent differentiation ability into chondrogenic, osteogenic, and adipogenic lineages^7^. hFCPCs express most MSC markers and show a clearer stem cell phenotype than that of MSCs. There are other reports describing similar stem or progenitor cell properties of hFCPCs at 6–20 weeks of gestation but their immune-phenotypic characteristics are not provided in these studeis^31,32^. Human chondrocytes from neocartilage were previously shown to have immune-privileged and immune-modulatory activities. However, chondrocytes are different from our fetal progenitor cells with stem cell properties, and information on Tregs and cytokine profiles was not provided^33^. We have previously shown that hFCPCs do not induce severe immune rejection when injected into the synovial cavity of rats^34^. In the present study, we characterized the immunological properties of hFCPCs from fetuses at 11–13 weeks of gestation and investigated their immune-modulatory activity on allogeneic lymphocyte proliferation. We found that hFCPCs share many characteristics with other fetal progenitor cells, as well as MSCs, but that they also display features unique to this cell type.

## Materials and Methods

### Culture of hFCPCs and MSCs

The use of human fetal tissues and bone marrow aspirate was approved by the institutional review board (IRB) of Ajou University Medical Center (AJIRB-MED-SMP-11-205). Cartilages from three fetuses between 11 and 13 weeks of gestation were obtained from donors undergoing elective termination with written and informed consent. Human fetal cartilage tissue has a large number of progenitor cells evenly distributed throughout the tissue^7^. The fetal cartilage tissue was carefully separated from femoral head with a scalpel blade, chopped into small pieces, and treated with 0.2% collagenase type II (Worthington Biochemical, Lakewood, NJ, USA) in serum-free Dulbecco’s modified Eagle medium (DMEM-LG; Hyclone, Logan, UT, USA) for 3 h at 37°C. Dissociated cells were collected by centrifugation at 1700 rpm for 10 min. After several washes with DMEM, cells were plated in a 150-mm culture plate at 8000 cells/cm^2^ and cultivated in DMEM-LG supplemented with 10% fetal bovine serum (FBS), 10,000 U/ml penicillin, and 0.1 mg/ml streptomycin (all from Hyclone). The medium was changed twice a week. Cells were passaged at 80% confluence using trypsin/EDTA (Invitrogen, Carlsbad, CA, USA). MSCs were isolated from the bone marrow of fractured femurs from patients 11–25 years old undergoing orthopedic surgery. Briefly, mononuclear cells were collected by Ficoll-Paque PLUS (GE Healthcare Bio-Sciences AB, Sweden) density gradient centrifugation, suspended in α-modified Eagle’s medium (α-MEM) supplemented with 10% FBS, 10,000 U/ml penicillin, and 0.1 mg/ml streptomycin (all from Hyclone), and plated at a density of 80,000 cells/cm^2^. After 6 days, non-adherent cells were removed, and the adherent MSCs were supplied with fresh medium. MSCs were passaged at 80% confluence using trypsin/EDTA, and plated at a density of 8,000 cells/cm^2^.

### Reverse Transcriptase-Polymerase Chain Reaction

Total RNA was isolated from hFCPCs using Tri-reagent (Invitrogen) according to the manufacturer’s instructions. cDNA synthesis was performed using a First Strand cDNA Synthesis Kit (Roche Diagnostics, Rotkreuz, Switzerland), and PCR was conducted using 1 μg cDNA, the primer pairs listed in Table 1, and HiPi PCR Premix (ELPis Biotech, Daejeon, Korea). Glyceraldehyde 3-phosphate dehydrogenase (GAPDH) was used as an internal control.

**Table 1.** Primers Used in the Present Study.

Gene	Primer Sequence (5′ – 3′)	Annealing temperature	Size	Accession Number
HLA-ABC	GTATTTCTTCACATCCTGGTCCCG GTCCGCCGCGGTCCAAGAGCGCAG	70	394	NM_002164.5
HLA-DR	CTGATGAGCGCTCAGGAATCATTG TGCATTGGCCAACATAGCTG	60	220	NM_001242758.1
HLA-DM	CCAGCCCAATGGAGACTG CAGCCCAGGTGTCCAGTC	57	136	NM002118.4
HLA-G	CTGACCCTGACCGAGACCTGG GTCGCAGCCAATCATCCACTGGAG	65	331	NM_002127.5
Beta2M	GTGGAGCATTCAGACTTGTC AACAAGCTTTGAGTGCAAGAG	57	479	NM_004048.2
CD80	ACTCGCATCTACTGGCAAAAGGA ATGGGAGCAGGTTATCAGGAAAA	59	553	NM_005191.3
CD86	GTATTTTGGCAGGACCAGGA GCCGCTTCTTCTTCTTCCAT	57	664	NM_006889.4
CD40	AGAAGGCTGGCACTGTACGA CAGTGTTGGAGCCAGGAAGA	59	363	NM_152854.2
TAP1	TCTCCTCTCTTGGGGAGATG GAGACATGATGTTACCTGTCTG	58	273	NM000593.5
TAP2	GTCGTGTCATTGACATCCTG TCAGCTCCCCTGTCTTAGTC	57	228	NM_000544.3
IDO	CGCTGTTGGAAATAGCTTC CAGGACGTCAAAGCACTGAA	53	234	NM_002164.5
LIF	GGCCCGGACACCCATAGACG CCACGCGCCATCCAGGTAAA	53	455	NM_002309.4
TGF-β	ACCGGCCTTTCCTGCTTCTCA CGCCCGGGTTATGCTGGTTGT	63	288	NM_000660.4
TSG-6	GGTGTGTACCACAGAGAAGCA GGGTTGTAGCAATAGGCATCC	63	284	NM_007115.3
sHLA-G	CCACCACCCTGTCTTTGACT TGGCACGTGTATCTCTGCTC	63	210	NM_002127.5
HGF	ATGCATCCAAGGTCAAGGAG TTCCATGTTCTTTTGTCCCACA	55	249	NM_001010932
GAPDH	GGTCATGAGTCCTTCCACGAT GGTGAAGGTCGGAGTCAACGG	58	520	NM_002046.3

### Flow Cytometry for Surface Marker Analysis

For the surface marker analysis, hFCPCs at passage four were untreated or stimulated with 200 U/ml of recombinant human IFN-γ (BD Biosciences, San Jose, CA, USA) for 4 days, and then incubated with fluorescence-conjugated primary antibodies against HLA-ABC, HLA-DR, CD80, CD86, CD40, CD40L, and CD11c (all from BD Biosciences) for 30 min at 4°C. After washing, the stained cells were analyzed on a BD FACSCanto II flow cytometer using Cell Quest software (BD Biosciences).

### Mixed Lymphocyte Reaction

Peripheral blood was obtained from five healthy volunteers (median age 39.3 years, range 31–46 years) with informed consent and IRB approval from Ajou University Medical Center (AJIRB-MED-SMP-11-205). Lymphocytes were isolated by Ficoll-Paque PLUS (1.077 g/ml; GE Healthcare Bio-Sciences AB, Sweden) density gradient centrifugation according to the manufacturer’s instructions. Peripheral blood lymphocytes (PBLs) at a concentration of 10^5^ cells in 0.5 ml medium were untreated or stimulated with 10 μg/ml concanavalin A (Con A; Sigma-Aldrich, St. Louis, MO, USA) for use in the MLR assay. hFCPCs or hMSCs were irradiated with 3000 rads Cs^137^ as previously described^35^ and plated at decreasing amounts of 10^5^, 10^4^, 10^3^, and 10^2^ cells in 96-well plates and mixed with 10^5^ Con A-stimulated or -unstimulated PBLs. After 4 days, 0.1 ml from each sample was transferred to a new 96-well plate, and 20 µl of BrdU labeling solution (BrdU ELISA kit, Roche Diagnostics, Mannheim, Germany) was added to each well. After 6 hours at 37°C, absorbance were measured at 492 nm using a microplate reader (Infinite m200; TECAN, Männedorf, Switzerland).

### Enzyme-Linked Immunosorbent Assays for Cytokines Analysis

PBLs (10^6^ cells) were stimulated with 10 μg/ml Con A and incubated with 10^5^ or 10^6^ Cs^137^-inactivated hFCPCs in 12-well plates. After 4 days, the culture medium was collected and the amount of IFN-γ, TNF-α, and IL-10 in samples was measured using enzyme-linked immunosorbent assay (ELISA) kits for each cytokine (eBioscience, San Diego, CA, USA) according to the manufacturer’s instructions. Absorbance at 450 nm was measured using a microplate reader (Infinite m200; TECAN).

### Analysis of Tregs

PBLs (10^6^ cells) were stimulated with 10 μg/ml Con A and incubated with Cs^137^-inactivated hFCPCs at a 1:1 ratio, as above, in a 60-mm dish for 4 days. PBLs were harvested after 4 days and immuno-stained simultaneously with fluorescence-conjugated anti-CD4-FITC, anti-CD25-APC, and anti-FoxP3-PE antibodies using a FoxP3 staining Kit (BD Biosciences) according to the manufacturer’s protocol. Cells were analyzed on a BD FACSCanto II flow cytometer using Cell Quest software (BD Biosciences).

### Statistical Analysis

Data were expressed as the mean ± standard deviation (SD) from multiple independent experiments, as indicated for each assay. Statistical significance was determined by one-way analysis of variance (ANOVA) with Tukey’s post hoc test using GraphPad Prism version 6.0 software (GraphPad Software, Inc., La Jolla, CA, USA). Values of *P* < 0.05 were regarded as statistically significant. Statistical significance was assigned as *P < 0.05, **P < 0.01, or ***P < 0.001.

## Results

### Immuno-Phenotypic Characterization of hFCPCs

**Fig 1.**
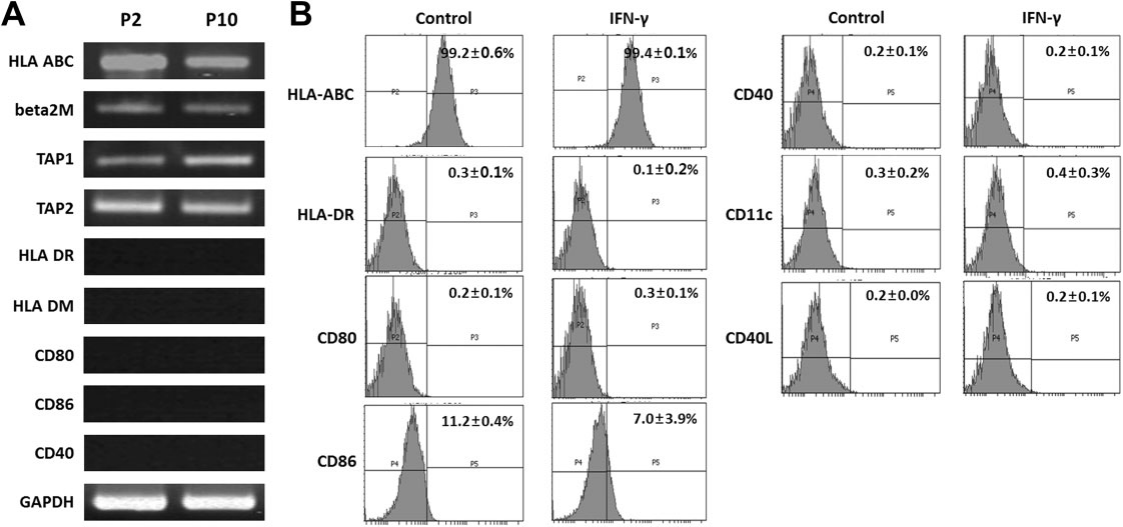
Immuno-phenotypic profiles of hFCPCs. (A) The expression of human immune-related genes was examined by RT-PCR analysis in hFCPCs at passages 2 (P2) and 10 (P10). The level of GAPDH mRNA were used as an internal control. (B) The expression of immune-related surface markers was examined in hFCPCs at passage 4 by flow cytometry. Cells were treated or untreated with IFN-γ for 4 days before analysis. In each panel, percentages of immune-positive cells are indicated by mean values with standard deviations (SD) from three independent experiments.

Expression of HLA class I and II and complement molecules in hFCPCs was examined by reverse transcriptase-polymerase chain reaction (RT-PCR) and flow cytometry. In the RT-PCR analysis, selected genes exhibited three different expression patterns at passages 2 and 10 of hFCPCs (Fig. 1A). HLA-ABC was expressed at high levels at passage 2, and expression decreased at passage 10. Beta-2 microglobulin (β2 M) and transporter associated with antigen processing molecules 1 (TAP1) and 2 (TAP2) were expressed at a consistent level at passages 2 and 10. HLA-DM, HLA-DR, CD80, CD86, and CD40 were not expressed at either passage. Flow cytometry was performed to examine the levels of selected immune-related antigens at passage 4 in the absence or presence of IFN-γ (Fig. 1B). HLA-ABC was present in 99.2 ± 0.6% of FCPCs, and CD86 was present only in 11.2 ± 0.4% of cells, while other cell surface markers, i.e. HLA-DR, CD80, CD40, CD11c, and CD40L were detected in less than 1% of cells. IFN-γ treatment is known to increase expression of HLA and complement molecules in MSCs^36^, but treatment with this cytokine but did not affect their expression in FCPCs. In the case of CD86, expression was decreased slightly to 7.0 ± 3.9% following IFN-γ treatment.

### hFCPCs Show Immune-Modulatory Activity in an MLR

**Fig 2.**
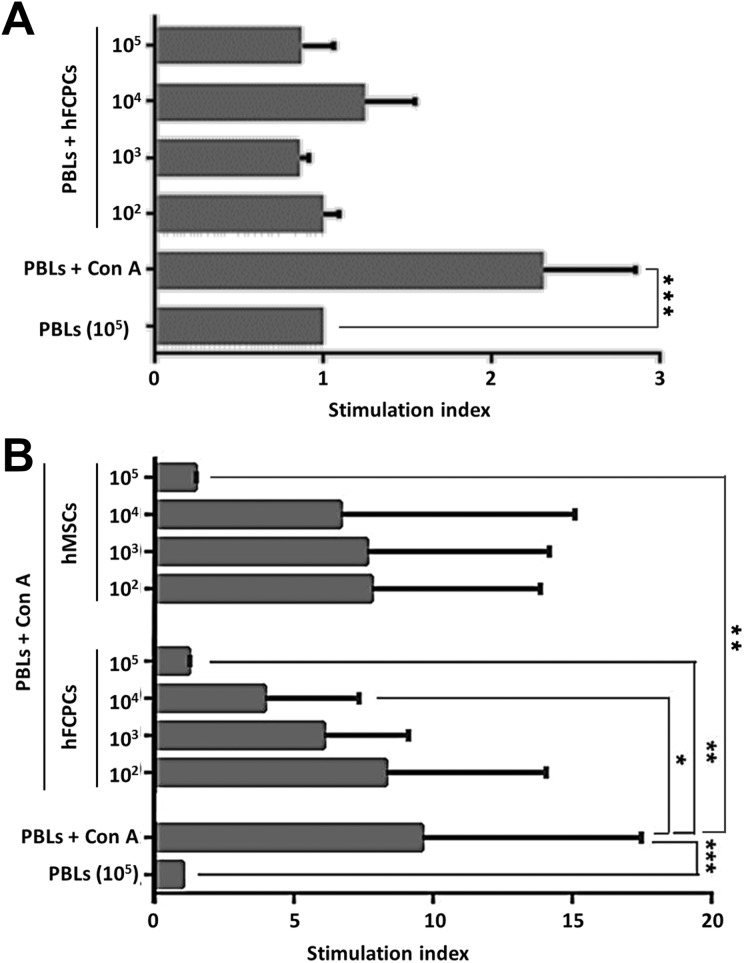
Effects of hFCPCs on the proliferation of allogeneic PBLs. (A) hFCPCs were irradiated with 3000 rads of Cs^137^ to abolish cell proliferation. Human PBLs (10^5^ cells) were cultured in 96-well plates in the absence or presence of inactivated hFCPCs at ratios of 1:1 and 1:1000 (10^5^ to 10^2^ cells) for 4 days. Human PBLs treated with 10 μg/ml Con A was used as a positive control. (B) Human PBLs (10^5^ cells) stimulated with Con A were co-cultured with inactivated hFCPCs or BM-MSCs at ratios of 1:1 and 1:1000 (10^5^ to 10^2^ cells) for 4 days. The proliferation of PBLs was measured by BrdU labeling and subsequent ELISA at 492 nm using a BrdU ELISA kit (Roche Diagnostics). Fold inductions from the values of untreated PBLs are shown in the histograms by mean values with SD from independent experiments (A, *n* = 3; B, *n* = 8 for hFCPCs, and *n* = 3 for MSCs). *P < 0.05, **P < 0.01, and ***P < 0.001.

MLR was performed using allogenic PLBs to understand the immune-stimulatory or -modulatory function of FCPCs. T cell proliferation by mitogens such as Con A has been regarded as mimicking T cell activation, and, therefore, has been used to establish positive controls. When hFCPCs were growth arrested and mixed with 10^5^ PBLs for 4 days at decreasing amounts of 10^5^, 10^4^, 10^3^, and 10^2^ cells, they did not stimulate T cell proliferation at all concentrations (Fig. 2A). Treatment of PBLs with 10 μg/ml Con A showed T cell proliferation of approximately 2.3 ± 0.5-fold (***P < 0.001). MSCs are known to suppress the immune response of PBLs stimulated with Con A^17^. We investigated whether hFCPCs show similar effects. When growth-arrested hFCPCs were mixed with Con A-stimulated PBLs under the same conditions, the hFCPCs inhibited T cell proliferation by Con A in a dose-dependent manner (Fig. 2B). When compared with Con A-stimulated PBLs alone, the effects of 10^5^ and 10^4^ hFCPCs were statistically significant (**P < 001 and *P < 0.05, respectively). In particular, 10^5^ hFCPCs mixed with PBLs at a 1:1 ratio almost completely blocked T cell proliferation in response to Con A. In this set of experiments, Con A induced relatively high levels of T cell proliferation with large variation (9.6 ± 7.9 fold) and showed a statistically significant difference from levels of proliferation of untreated PBLs (***P < 0.001). For comparison, hMSCs from bone marrow also inhibited Con A-induced T cell proliferation, but this effect was not greater than that of hFCPCs. The effect was statistically significant only at 10^5^ hFCPCs (***P < 0.001), with high variation observed with less hFCPCs. In a separate experiment, we investigated the immune-modulatory activity of human young (1 year old) and adult chondrocytes (56 years old) and found they did not efficiently suppress PBL activation by Con A (Supplementary Fig 1). Interestingly, we found that adult chondrocytes further increased proliferation of PBLs.

### hFCPCs Modulate Cytokine Production from PBLs

**Fig 3.**
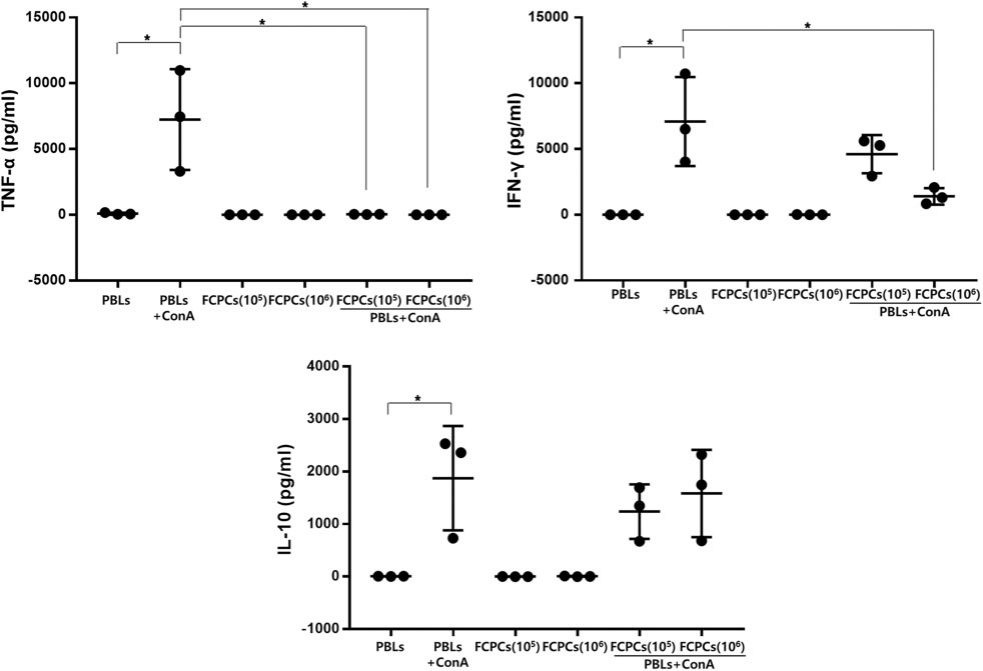
Effect of hFCPCs on the expression of inflammation-related cytokines in PBLs stimulated with Con A. PBLs (10^6^ cells) were stimulated with 10 μg/ml Con A in the absence or presence of inactivated hFCPCs (10^5^ or 10^6^ cells) for 4 days. Untreated PBLs and inactivated hFCPCs were used as controls. The culture media were collected, and TNF-α, IFN-γ, and IL-10 levels were determined by ELISA (eBioscience). The graphs shows individual values and means with SD from three independent experiments. The statistical significance of differences between the PBL + Con A and PBL + Con A groups and the hFCPCs groups was assessed using a paired t test. *P < 0.05, **P < 0.01, and ***P < 0.001.

To determine whether hFCPCs affect the cytokine profiles of activated PBLs, 10^5^ or 10^6^ hFCPCs were co-cultured with 10^6^ Con A-treated PBLs for 4 days, and expression of TNF-1α, IFN-γ, and IL-10 was examined (Fig. 3). TNF-1α and IFN-γ are known to be pro-inflammatory cytokines, whereas IL-10 is an anti-inflammatory cytokine^37^. The Con A-treated samples showed substantial variation, and the data from all three samples were presented together with mean values ±SD. In spite of individual differences, Con A significantly increased secretion of TNF-α from 85.4 ± 71.4 to 7242.8 ± 3838.8 pg/ml, IFN-γ from 5.4 ± 9.3 to 7087.0 ± 3385.7 pg/ml, and IL-10 from 4.4 ± 2.4 to 1874.6 ± 993.0 pg/ml. Co-culture of 10^5^ or 10^6^ hFCPCs with 10^6^ Con A-treated PBLs almost completely abolished TNF-α secretion (36.6 ± 9.1 and 3.4 ± 1.4 pg/ml, respectively) and significantly reduced IFN-γ secretion to 4606 ± 1448.5 and 1404.8 ± 623.4 pg/ml, respectively. In contrast, co-culture with these cells did not hamper Con A-induced IL-10 secretion significantly, with levels of IL-10 secretion of 1238.3 ± 518.6 and 1584.5 ± 831.7 pg/ml, respectively.

### hFCPCs Increase the Number of Tregs in PBLs

**Fig 4.**
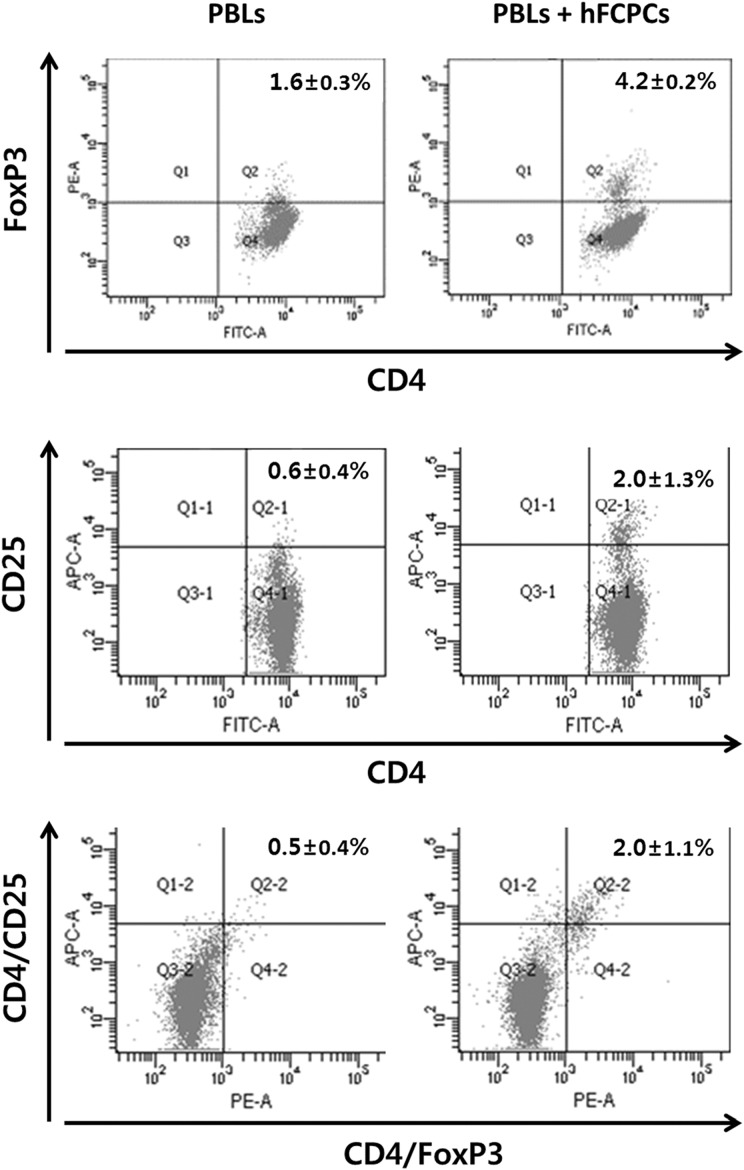
Effects of hFCPCs on the activation of CD4^+^CD25^+^FoxP3^+^ cells in PBLs. Human PBLs were cultured with inactivated hFCPCs for 4 days and subjected to triple staining with anti-human CD4-FITC, CD25-APC, and FoxP3-PE using a FoxP3 staining kit (BD Bioscience). The graphs show representative flow cytometry data that indicate the subpopulations of CD4^+^CD25^+^, CD4^+^FoxP3^+^, and CD4^+^/CD25^+^/FoxP3^+^ cells. Percentages of double- or triple-positive cells were calculated from four independent experiments and are presented in the upper right quadrant (Q2) as the means with SD (*n* = 4).

CD4^+^CD25^+^ Treg cells are capable of modulating tolerance to immune responses^38^, and Foxp3, another Treg cell marker, can control Treg development^39^. Growth-arrested hFCPCs were co-cultured with unstimulated PBLs at a 1:1 ratio for 4 days, and the number of CD4^+^CD25^+^Foxp3^+^ T cells was determined by flow cytometry (Fig. 4). Combining two markers, co-culture with hFCPCs increased the amount of CD4^+^Foxp3^+^ cells from 1.6 ± 0.3% to 4.2 ± 0.2%, that of CD4^+^CD25^+^ cells from 0.6 ± 0.4% to 2.0 ± 1.3%, and that of FoxP3^+^CD25^+^ cells was from 0.5 ± 0.4% to 2.0 ± 1.1%). In addition, the percentage of CD4^+^CD25^+^ Foxp3^+^ T cells of total PBLs was 0.5 ± 0.4%, but this percentage was increased after co-culture with hFCPCs (2.0 ± 1.1%).

### hFCPCs Express Many Immune-Modulatory Factors

**Fig 5.**
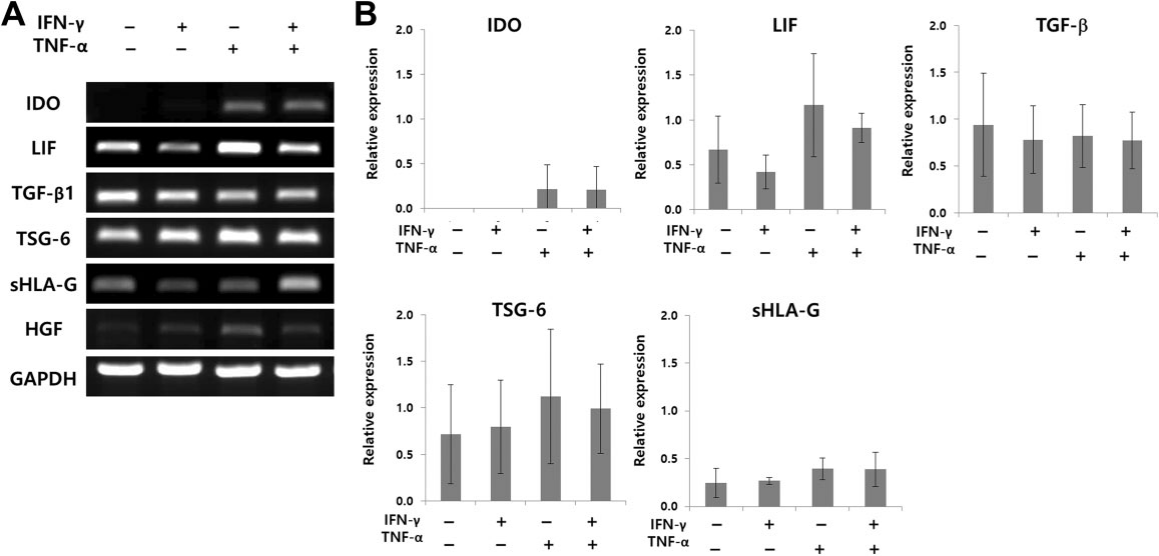
Expression of selected cytokines in hFCPCs stimulated with IFN-γ and/or TNF-α. hFCPCs from three donors were treated with 500 U/ml of TNF-α, 200 U/ml of IFN-γ, or their combination for 12 h. (A) A representative data on the mRNA levels of IDO, LIF, TGF-β, TSG-6, HLA-G, and HGF in hFCPCs examined by RT-PCR. GAPDH was used as an internal control. (B) RT-PCR data from three different donors were quantified using Image J program. Relative expression of each cytokine normalized to that of GAPDH are presented by mean values with SD (*n* = 3).

The effect of MSCs on T cells is modulated mainly through cell contact-independent processes, indicating the importance of soluble factors such as IDO, LIF, TGF-β1, sHLA-G5, and HGF^15^. Expression of these factors in MSCs is induced by IFN-γ and TNF-α^40^. hFCPCs were untreated or treated with IFN-γ and/or TNF-α for 12 h, and the expression levels of these factors were measured by RT-PCR (Fig. 5A). Untreated hFCPCs showed high levels of LIF, TGF-β, and TSG-6 expression, low levels of sHLA-G5 expression, and no evident IDO and HGF expression. Treatment with INF-γ did not increase expression of these factors and only slightly decreased expression of LIF and HLA-G. Treatment with TNF-α clearly increased IDO, TSG-6, and HGF expression. Co-treatment with INF-γ and TNF-α showed mixed results, with only IDO and sHLA-G5 expression increased when compared with that of the untreated control. Quantitative data of the RT-PCR results showed similar patterns to the representative image but the statistical significance was not observed due to the large variation among three different donors (Fig. 5B). The graph on HGF is not included because its band intensity was too weak to obtain meaningful quantification.

## Discussion

This study is the first to define the immune-privileged and immune-modulatory characteristics of hFCPCs *in vitro*. We found that hFCPCs express HLA class I and associated molecules but do not express HLA class II molecules or co-stimulatory factors such as CD80 (B7 -1), CD86 (B7-2), and CD40. HLA class I molecules are expressed in almost all mammalian cells, whereas class II molecules are mainly found in antigen presenting cells (APCs) such as dendritic cells (DCs). HLA molecules and co-stimulatory factors are mainly responsible for antigen presentation and immune responses^36^. Cells that express HLA molecules stimulate T cells directly if co-stimulatory factors such as CD80 or CD86 are also present; otherwise, they activate T cells indirectly by cross presentation of their peptides on APCs such as DCs^36^. Therefore, cells without HLA molecules might have a low risk of alloimmunity. hFCPCs are expected not to express co-stimulatory molecules, and, therefore, may not directly activate T cells. Because direct activation of T cells is known to be about 10-fold stronger than activation through the indirect APC-dependent mechanism^41^, the allogenic immune response of hFCPCs themselves might not be a significant problem, as confirmed by our MLR assay. Although there should be a mechanistic difference between the allogeneic and xenogeneic immune rejection, these findings might be in connection with our previous report that hFCPCs did not cause immune rejection in rat synovial cavity^34^. Previously, adult or fetal MSCs were shown to express HLA class I molecules without expressing HLA class II or co-stimulatory molecules^14,16^. However, the expression of both HLA class I and II molecules on cell surfaces increased when cells were exposed to INF-γ for 7 days. Similarly, expression of HLA molecules increased on the surfaces of fetal and adult MSCs in response to IFN-γ^14^. In contrast to fetal or adult MSCs, treatment with IFN-γ for 4 days did not increase HLA molecules of either type on the surfaces of hFCPCs, as demonstrated by flow cytometry. A previous report has shown that chondrocytes isolated from human iPSC-derived cartilages express low levels of both HLA types I and II, and treatment of IFN- γ induces expression of HLA type I only^42^. Taken together with our findings using hFCPCS, it could be speculated that chondrocytes have an innate property of low expression of HLA type II and no significant induction of its expression upon IFN-γ stimulation.

MSCs from the bone marrow can suppress alloreactive T cells^43^. They also significantly inhibit proliferation of T cells stimulated by potent mitogens such as Con A and phytohemagglutinin (PHA). This effect of mitogens is regarded as similar to that of T cell activation by APCs^44^. We found in this study that the immunosuppressive effect of hFCPCs is stronger than that of adult MSCs. hFCPCs almost completely blocked Con A-induced proliferation of allogeneic PBLs when they were mixed at a 1:1 ratio. Our preliminary *in vitro* data also shows that TNF-α but not IFN-γ induced the expression of anti-inflammatory factors, including IDO, LIF, TSG-6, and HGF, in hFCPCs. Interestingly, we found that both young (1 year) and aged (56 years) chondrocytes showed no clear immunosuppressive activity in MLR (supplementary Fig 1), while human iPSCs-derived chondrocytes were reported to have an immunosuppressive activity^42^. These findings suggest that the immunosuppressive activity is specific to pre-natal chondrocytes and the iPSCs-derived chondrocytes might share their characteristics.

To better understand the immunosuppressive effects of hFCPCs, we measured the secretion of INF-γ, TNF-α, and IL-10 in Con A-activated PBLs in the presence of hFCPCs. IFN-γ and TNF-α are key players in allogeneic immune responses and T cell proliferation. IFN-γ induces expression of HLA class I and II molecules on cell surfaces and reinforces T cell activity^45^. TNF-α is produced by monocytes or macrophages and enhances proliferation of mature T cells^46^. Our results indicate that hFCPCs can suppress expression of these two inflammatory cytokines in Con A-activated PBLs. This result agrees with previous reports on different types of MSCs from bone marrow, Wharton’s jelly, or umbilical cord blood^47,48^. IL-10 is a potent immunosuppressive cytokine *in vitro* and *in vivo* that downregulates production of pro-inflammatory cytokines and chemokines by immune cells^49^. Previously, bone marrow MSCs were shown to induce IL-10 expression in MLR assays, particularly when primed with IFN-γ or TNF-α before co-culture with PBLs^47^. Interestingly, in our study, hFCPCs did not increase IL-10 expression in either resting or Con A-activated PBLs in the MLR assay. Similar results have been reported for MSCs from bone marrow, which did not increase IL-10 expression in either suppressed and unsuppressed conditions in an MLR assay^36^. Taken together, these results suggest that MSCs and hFCPCs shows various effects on cytokine section from PBLs, depending on their specific cell type and source, which might affect their immune-modulatory activities.

Numerous reports have shown the importance of CD4^+^CD25^+^Foxp3^+^ Tregs in immune regulation. Tregs have pleiotropic suppressive effects on immune responses to allo-antigens and infectious agents^50^ and play a key role in immune tolerance of a fetus to its mother^28^. MSCs modulate immune responses by *de novo* induction and expansion of CD4^+^CD25^+^Foxp3^+^ and CD8^+^ Tregs, which can promote immune tolerance in certain circumstances^51^. Our results showed a 2.63-fold increase in the number of CD4^+^Foxp3^+^ Tregs in PBLs co-cultured with hFCPCs, which is significant when compared with previous data for MSCs. Induction of Tregs by MSCs is mediated by direct contact and indirectly through CD4^+^ T cells via TGF-β, IDO, HLA-G5, LIF, and PGE2 secreted by MSCs^25,52^. We found that hFCPCs also express TGF-β, LIF, and HLA-G5, which suggests that expression of these cytokines from hFCPCs may play a role in expanding the Treg population.

The immunosuppressive effect of MSCs can be mediated in a cell-cell contact-independent manner. Soluble factors such as TGF-β1, HO-1, PGE2, IDO, HLA-G5, LIF, TSG-6, and HGF are known to participate in this process^15^. hFCPCs in this study expressed LIF, TGF-β1, and TSG-6 at high levels and HLA-G at moderate levels, whereas they did not express IDO and HGF. Treatment of hFCPCs with TNF-α induced expression of IDO, LIF, TSG-6, and HGF slightly, but treatment with IFN-γ showed almost no effect on expression of these genes. IDO is an enzyme that catabolizes tryptophan into kynurenine, which regulates T cell proliferation^53^. IDO is expressed in MSCs, and its expression has been shown to increase significantly in response to IFN-γ^23^, which is different from what was observed with hFCPCs. Expression of LIF increases in response to various inflammatory insults, such as exposure to lipopolysaccharide, IL-6, IL-1β, or G-CSF^54^. MSCs also express LIF, and expression of this factor is further induced by interactions between MSCs and PBLs, which play a role in the proliferation of Foxp3^+^ Tregs^19^. HLA-G is an important factor that mediates the immunosuppressive function of MSCs; in particular, it inhibits the innate immune responses of natural killer (NK) cells and secreted IFN-γ^25^. TGF-β1 also contributes to the immunosuppressive function of MSCs and is probably induced by direct contact between MSCs and monocytes^55^. Specifically, TGF-β1 secreted by MSCs induces Tregs and suppresses T cell responses. Overall, the secretion of these immune- and inflammation-modulating factors by hFCPCs is similar to that of MSCs, but there are clear differences as well particularly in that hFCPCs did not respond to IFN-γ. Further studies are needed to clarify the secretome profile and mechanisms underlying the immune-modulatory functions of hFCPCs.

The results of this study strongly suggest the therapeutic potential of hFCPCs to treat immune problems or inflammatory diseases. We have previously tried to inject hFCPCs into the synovial cavity of rats with Complete Freund’s Adjuvant-induced knee arthritis and compare its therapeutic effect with that of triamcinolone (TRA), a representative anti-arthritis drug^34^. We found in the study that hFCPCs was not highly efficient but reduced the knee circumference at a delayed time point 7 days after injection with no immune rejection or serological toxicity. It is not clear if this result has implications for the therapeutically effective dose of hFCPCs needed in physiological or other disease environments *in vivo*. However, hFCPCs appear not to be inferior to MSCs in their immunosuppressive activity, detailed in many previous reports^47,48,52^. Therefore, we speculate that the previous result suggests both clinical possibility of hFCPCs and needs for further optimization in the target indications and therapeutic protocol. The behavior and mechanism of hFCPCs in an inflammatory environment *in vivo*, including whether these cells would have a significant therapeutic benefit, should be explored in a future study.

Stem or progenitor cells of fetal origin have been regarded as a good source of therapeutic transplantation^56^. Fetal cells are know to have high proliferation ability and high differentiation ability to the committed lineages. We have also revealed that hFCPCs have proliferation ability and produce high quality cartilage tissue both *in vitro* and *in vivo*^7^. Although the availability of donor tissue is limited, a fetal cartilage yields more than 20-fold the number of cells than the same amount of adult cartilage. In addition, we have also shown that hFCPCs can be expanded for more than 30 passages without losing their proliferation ability in a xeno-free medium, which resulted in approximately 10^30^ cells from 5 × 10^5^ cells of initial culture^57^. Therefore, we believe hFCPCs have a competitive commercial value, but understand that actual commercialization requires further investigation on their characteristics and behavior during long-term expansion and development of means for mass production and quality control as well. Lastly, there are ethical concerns with using cells of fetal origin, although it is not absolutely illegal and there are many clinical trials underway using fetal stem/progenitors^57^. We think the balance between the ethical concerns and patient benefits should be considered as well.

In conclusion, the results of this study revealed that hFCPCs have immune-privileged and immune-modulatory characteristics similar to those of MSCs. The mechanisms underlying these hFCPCs functions also appear to be similar to those of MSCs, but the secretion of immune-modulatory factors and the response to inflammatory cytokines of TNF-α and IFN-γ are distinct between these two cell types. Together with the advantages of their high proliferation ability, stem cell properties, and low safety concerns, the immune-privileged and immune-modulatory functions of hFCPCs suggest their utility in diverse applications and high therapeutic value in regenerative medicine.

## Supplemental Material

Supplementary_Figure_1 - Immunophenotype and Immune-Modulatory Activities of Human Fetal Cartilage-Derived Progenitor Cells Click here for additional data file. Supplementary_Figure_1 for Immunophenotype and Immune-Modulatory Activities of Human Fetal Cartilage-Derived Progenitor Cells by Su Jeong Lee, Jiyoung Kim, Woo Hee Choi, So Ra Park, Byung Hyune Choi and Byoung-Hyun Min in Cell Transplantation
