# Supplementary material for: Immunophenotype and Immune-Modulatory Activities of Human Fetal Cartilage-Derived Progenitor Cells
Source: Cell Transplant. 2019 Apr 14;28(7):932–42. doi: 10.1177/0963689719842166 (PMC6719489; doi:10.1177/0963689719842166)
Supplement: Supplementary_Figure_1 - Immunophenotype and Immune-Modulatory Activities of Human Fetal Cartilage-Derived Progenitor Cells [file Supplementary_Figure_1.pdf]

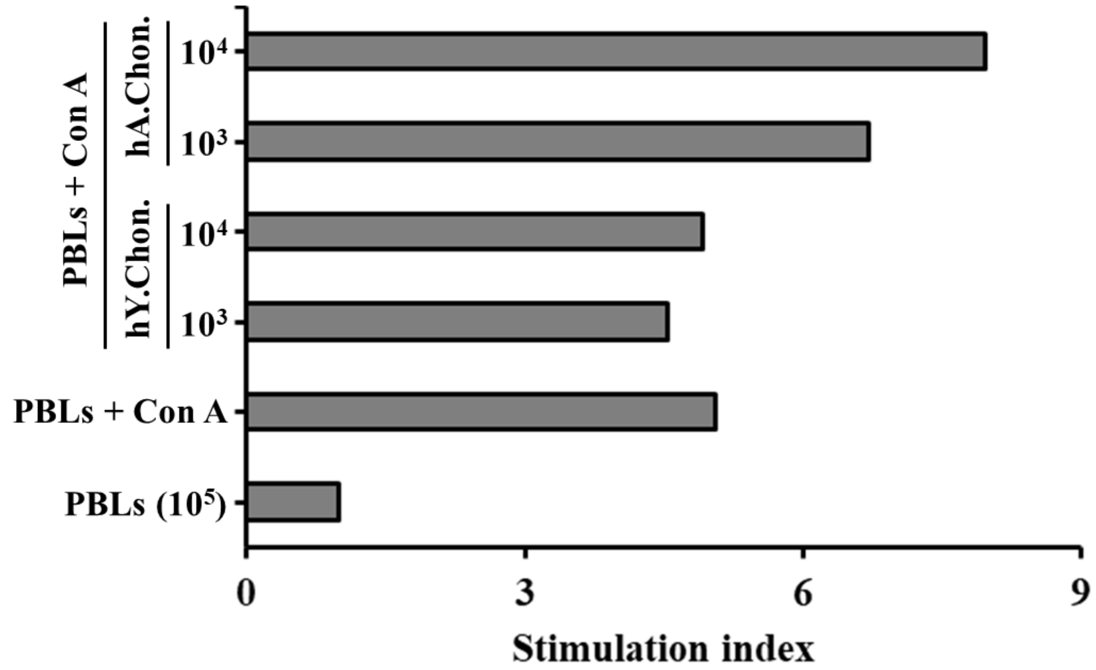

Supplementary Figure 1. Effect of human young and adult chondrocytes on the ConA-mediated activation of allogeneic lymphocytes. Human young chondrocytes (hY.Chon) and adult chondrocytes (hA.Chon) were irradiated with 3,000 reds Cs^{137} to abolish cell proliferation. Human peripheral blood lymphocytes (PBLs) were cultured in 96-well plates at 10^5 cells per well and stimulated with μg/ml Concanavalin A (Con A). PBLs treated with Con A were untreated or treated with irradiated hY.Chon or hA.Chon at 1:10 and 1:100 ratios (10^4 and 10^3 cells) for 4 days. The proliferation of PBLs was measured by BrdU labeling and subsequent ELISA at 492 nm using a BrdU ELISA kit (Roche Diagnostics). Fold inductions from the values of untreated PBLs are shown in the histograms.
